# *Alcaligenes ammonioxydans* HO-1 antagonizes *Bacillus velezensis via* hydroxylamine-triggered population response

**DOI:** 10.3389/fmicb.2022.920052

**Published:** 2022-07-22

**Authors:** Xi-Yan Gao, Wei Xie, Ying Liu, Lan Ma, Zhi-Pei Liu

**Affiliations:** ^1^State Key Laboratory of Microbial Resources, Institute of Microbiology, Chinese Academy of Sciences, Beijing, China; ^2^College of Life Science, University of Chinese Academy of Sciences, Beijing, China

**Keywords:** *Alcaligenes ammonioxydans* HO-1, antagonism, hydroxylamine, *Bacillus velezensis* V4, autolysis, population response

## Abstract

Antagonism is a common behavior seen between microbes in nature. *Alcaligenes ammonioxydans* HO-1 converts ammonia to nitrogen under aerobic conditions, which leads to the accumulation of extracellular hydroxylamine (HA), providing pronounced growth advantages against many bacterial genera, including *Bacillus velezensis* V4. In contrast, a mutant variant of *A. ammonioxydans*, strain 2-29, that cannot produce HA fails to antagonize other bacteria. In this article, we demonstrate that cell-free supernatants derived from the antagonistic HO-1 strain were sufficient to reproduce the antagonistic behavior and the efficiency of this inhibition correlated strongly with the HA content of the supernatant. Furthermore, reintroducing the capacity to produce HA to the 2-29 strain or supplementing bacterial co-cultures with HA restored antagonistic behavior. The HA-mediated antagonism was dose-dependent and affected by the temperature, but not by pH. HA caused a decline in biomass, cell aggregation, and hydrolysis of the cell wall in exponentially growing *B. velezensis* bulk cultures. Analysis of differential gene expression identified a series of genes modulating multicellular behavior in *B. velezensis*. Genes involved in motility, chemotaxis, sporulation, polypeptide synthesis, and non-ribosomal peptide synthesis were all significantly downregulated in the presence of HA, whereas autolysis-related genes showed upregulation. Taken together, these findings indicate that HA affects the population response of coexisting strains and also suggest that *A. ammonioxydans* HO-1 antagonize other bacteria by producing extracellular HA that, in turn, acts as a signaling molecule.

## Introduction

Bacteria represent some of the most abundant life forms on earth. They rarely exist alone, and competition for scarce resources is common in diverse microbial communities. The ability to survive and divide in the presence of other strains and species is essential for evolutionary success (Granato et al., 2019) and leads to the development of multiple strategies to compete for resources and space (Lin et al., 2020). Antagonism is one of the most common phenomena in diverse microbial communities (Peterson et al., 2020), enabling bacteria to establish spatial and nutrient niches by inhibiting or killing their neighbors (Yim and Wang, 2021). With the emergence of (meta)genomic data, multiple mechanisms for bacterial antagonism have been discovered, including chemical, mechanical, and biological mechanisms (Granato et al., 2019; Klein et al., 2020). Almost all major bacterial phyla possess antagonistic pathways that may or may not require cellular contact (Peterson et al., 2020). The need to compete in order to survive drove the evolution of proteins, peptides, and small soluble molecules that can diffuse into surrounding environments and mediate interactions between bacterial cells (Yim and Wang, 2021).

Interbacterial antagonism can impact the assembly and stability of the microbiome and has the potential to modulate microbial communities in diverse environments (Yim and Wang, 2021). It has been proposed that some naturally occurring mechanisms of antagonism may be harnessed to suppress plant diseases in agriculture, offering an alternative to the use of chemicals (Perez-Garcia et al., 2011).

*Bacillus velezensis* and *Alcaligenes* species represent the most widely studied plant growth-promoting rhizobacteria (PGPR) that coexist in ecological and evolutionary dimensions (Lahlali et al., 2020). *B. velezensis* strains are widely distributed in nature with robust growth and survival characteristics. They are easy to isolate, cultivate, harmless to humans and animals, and do not pollute the environment. They produce a rich spectrum of metabolites, with broad-spectrum antibacterial activity and strong anti-stress action (Perez-Garcia et al., 2011; Gao et al., 2017; Lahlali et al., 2020; Dimkić et al., 2022). Many *B. velezensis* strains gained practical application in promoting plant growth and antagonizing pathogens (Fan et al., 2018). *Alcaligenes* species are another group of potential PGPR (Yokoyama et al., 2013; Ray et al., 2016; Gong et al., 2019; Lahlali et al., 2020), with distinct biotechnological potential in the pharmaceutical industry and bioremediation of contaminated environments (Ray et al., 2015; Dixit et al., 2020; Felestrino et al., 2020; Onajobi et al., 2020; Zouari et al., 2020; Fernandes et al., 2021).

*Bacillus velezensis* V4 is an extensively studied isolate with a pronounced ability to control biological contamination (Gao et al., 2017, 2022). Approximately 8.4% of the genome of the V4 isolate consists of genes involved in the synthesis of bioactive secondary metabolites, including peptides (e.g., bacilysin), lipopeptides (e.g., surfactins, iturins, fengycins), polyketides (e.g., macrolactin, bacillaene, difficidin/oxydifficidin), and siderophores (e.g., bacillibactin) (Gao et al., 2017). This abundance of secondary metabolites makes V4 a robust strain, enabling it to thrive in various environments.

Under aerobic conditions, *Alcaligenes ammonioxydans* HO-1 converts ammonia to N_2_. This direct ammonia oxidation pathway (Dirammox) encoded by the *dnfT1RT2ABCD* gene cluster leads to the accumulation of hydroxylamine (HA) (Wu et al., 2021).

During attempts to establish a diverse bacterial consortium for pathogen control in agricultural use, we observed a contact-independent antagonism between these two strains. This antagonism between HO-1 and V4 strains limited our ability to establish mixed consortia, reducing the efficacy of biological control provided. The importance of the potential beneficial effects provided by the presence of *B. velezensis* V4 in consortia prompted us to elicit the mechanism(s) underlying this antagonism.

Here, we report a close link between the accumulation of HA and antagonism observed in the combined cultures. These results provide new insights into the molecular basis and mechanism of contact-independent antagonism, highlighting the novel role of HA as a signaling molecule for bacterial antagonism.

## Materials and methods

### Bacterial strains and culture conditions

*Alcaligenes ammonioxydans* HO-1 (Wu et al., 2021) and 2-29 were used throughout the study. Strain 2-29 is a mutant of HO-1 that has lost the ability to produce HA as a result of random mutagenesis, using *N*-methyl-N′-nitro-*N*-nitrosoguanidine. The loss of function is due to a single base mutation causing an E^444^ to K substitution in *dnfR*. Since the *dnfT1RT2ABCD* gene cluster is transcriptionally regulated by *dnfR*, this point mutation renders the *dnfT1RT2ABCD* gene cluster non-expressed. Thus, 2-29 cells no longer have the capacity to produce HA. In complementation experiments, the 2-29 strain was transfected with the intact *dnf* R gene in a pBBRMCS-plasmid. *B. velezensis* V4 was used as the target strain. The used HO-1 and V4 strains were deposited in the China General Microbiological Culture Collection Center (CGMCC) under accession numbers CGMCC 1.16549 and CGMCC no. 10149, respectively.

The 40 additional bacterial strains used in this study are listed in Table 1. These were isolated from water environments or were already in storage in our laboratories.

**Table 1:** Antagonistic effect of *A. ammonioxydans* HO-1 against 40 environmental bacterial isolates.

**Genus**	**Species and strain**	**Source(s)**	**Antagonism (**+**/-)**
*Bacillus*	*B*. sp. HK-S11, *B*. sp. BFW1, *B*. *methylotrophicus* L7, *B*. *megaterium* HK-S1, *B*. *pumilus* H2, *B*. *flexus* HK-W2, *B. safensis* B3, *B. velezensis* XK, *B*. sp. HK-S12, *B*. sp. J7	Sediment, fresh water, marine water, or stored in Lab	+
*Pseudomonas*	*P*. sp. AOB-7, *P*. sp. YL12-1R, *P*. sp. A85, *P*. sp. YL12, *P*. sp. SUR, *P*. sp. 2-8, *P*. sp. RL13-3, *P*. sp. RL13-3, *P*. sp. RL13-4	Bioreactor or marine water	+
*Marinobacter*	*M*. sp. NNA5	Marine water	+
*Comamonas*	*C*. *aquatica* HK-W11, *C*. *faecalis* W9-1	Bioreactor	+
*Vibrio*	*V. vulnificus* CZ-A2, *V*. sp. LM2	Marine water	+
*Rhodococcus*	*R*. sp. RL13-1, *R*. sp. RL13-2	Soil	+
*Exiguobacterium*	*Exiguobacterium* sp. HK-W5	Fresh water	+
*Lysinibacillus*	*Lysinibacillus fusiformis* RL13-11	Bioreactor	+
*Escherichia*	*E. Coli* DH5α	Stored in Lab	+
*Enterobacter*	*Enterobacter* sp. FT3	Fresh water	+
*Arthrobacter*	*Arthrobacter soil* 2-C4	Soil	+
*Brevundimonas*	*Brevundimonas* sp. HK-W28	Fresh water	+
*Castellaniella*	*C. denitrificans* HK-W25	Bioreactor	+
*Alcaligenes*	*A. ammonioxydans* 2-29, *A*. sp. 1.1786, *A*. sp. 1-2.1, *A*. sp. 16503-2, *A*. sp. GL12, *A*. sp. PC01, *A*. sp. 1.0609	Bioreactor	-

*Alcaligenes ammonioxydans* strains were cultivated in LB or HNM medium (Wu et al., 2021), prepared according to Liu et al. (2016), but without NaCl. *B. velezensis* V4 was cultivated in minimum medium (MM), prepared according to HNM, but substituting sucrose instead of sodium succinate as the carbon source.

### Inhibition tests using co-cultures of *A. ammonioxydans* and *B. velezensis* V4

*Alcaligenes ammonioxydans* strains and *B*. *velezensis* V4 were incubated in LB/HNM broth for 24 h at 30°C with shaking (150 rpm), monitoring OD_600_ at 2 h intervals. For the inhibition test, 100 μl of the V4 culture was spread onto LB agar plates and a 6 mm sterile paper disk was placed in the center. Also, 10 μl of HO-1/2-29 LB/HNM culture was pipetted onto this disk and the plates were incubated at 30°C for 24 h, measuring the area of the inhibition zone at this time point.

In the co-culture experiments, the cell suspensions of all the three bacterial cultures were adjusted to OD_600_ 0.5 × 10^−2^. Cell suspensions were mixed at an OD_600_ ratio of 1:2, 1:1, and 2:1. Then, 100 μl of the mixed suspension was sprayed on an LB agar plate and cultured for 72 h at 30°C to verify and compare the interbacterial competitive advantage of HO-1 and 2-29 cells against V4.

To obtain cell-free supernatants (CFS) of HO-1 and 2-29 cultures, 1 ml aliquots were collected at hourly intervals. Cells were removed by centrifugation (8,000 × *g*) for 10 min at 4°C, followed by passing the supernatant through a sterile syringe filter. HA concentration in these CFSs was determined as detailed in the “Analytical methods” section. LB agar plates were sprayed with V4 cultures and 200 μl of the various CFS were added into Oxford Cup(s) placed on the plate. The area of the inhibition zone was measured after incubating the plate for 24 h at 30°C.

### The effect of HA concentration, temperature, and pH on antagonism

Solutions of HA at final concentrations of 0.2, 0.5, 0.8, 1.0, 2.0, and 5.0 mM were prepared in 0.9% NaCl. The inhibition tests were carried out as described in the “Inhibition tests using co-cultures of *A. ammonioxydans* and *B. velezensis* V4” section. In other experiments, 200 μl of 2.0 mM HA solution was applied and plates were incubated at 4, 15, 25, 30, 37, and 45°C. The effect of pH was tested by preparing a series of 2.0 mM HA solutions with the pH adjusted to 2.0, 3.0, 4.0, 5.0, 6.0, 7.0, 8.0, 9.0, and 10.0. The inhibitory effect of these solutions on the growth of *B. velezensis* V4 was tested as discussed earlier. In all these tests, the area of inhibition zone was measured.

### The effect of HA on the survival kinetics of *B. velezensis* V4

The survival kinetics of *B. velezensis* V4 exposed to various growth conditions were studied as follows. CFSs of V4, HO-1, and 2-29 cultures were prepared as described in the “Inhibition tests using co-cultures of *A. ammonioxydans* and *B. velezensis* V4” section and called V4CFS, HCFS, and UCFS, respectively. In addition, UCFS supplemented with 2.0 mM (termed UCFS-2.0 mM HA), V4CFS supplemented with 0.2, 0.5, 1.0, 2.0, and 5.0 mM of HA (termed V4CFS-0.2/0.5/1.0/2.0/5.0 mM HA), and 2.0 mM HA dissolved in 0.9% NaCl (termed 0.9% NaCl-2.0 mM HA) were all used to cultivate *B. velezensis* V4.

Exponentially growing cultures of V4 in LB broth, OD_600_ ~ 2.5–3.0, were pelleted, resuspended in V4CFS, HCFS, UCFS, UCFS-2.0 mM HA, and V4CFS 0.2-5.0 mM HA in 96-well plates and the OD_600_ was monitored at 20 min intervals using a CLARIOstar Plus (BMG LABTECH) micro-plate reader.

To investigate the morphology of V4 cells after treatment, cells grown in V4CFS, HCFS, UCFS, and V4CFS-2.0 mM HA were collected after 3 h incubation, resuspended in 2.5% glutaraldehyde solution in 0.1 M phosphate-buffered saline (v:v), and fixed for 24 h. Details of the processing and scanning electron microscopy (SEM, model S-3400N, Hitachi Instruments, Japan) were described in detail previously (Gao et al., 2017).

### The effects of HA on *B. velezensis* V4 colony morphology

To test the effect of HA on the morphology of typical V4 colonies, 100 μl V4 cell suspension (OD_600_ = 0.5 × 10^−2^) was spread onto LB plates as described in the “Inhibition tests using co-cultures of *A. ammonioxydans* and *B. velezensis* V4” section. The plates were incubated at 30°C for 24 h. At this point, 200 μl of 2.0 mM HA was added to an Oxford Cup placed near the V4 colonies and the plate was incubated for an additional 24 h. Colonies were photographed before and after HA treatment and differences were analyzed.

### Transcriptome profiling and quantitative PCR analysis

Exponentially growing V4 cells, 20 h in LB broth, were exposed to V4CFS (as control) or V4CFS-2.0 mM HA for 3 h. Cells were collected by centrifuging (8,000 rpm, 3 min at 4°C), snap-frozen in liquid nitrogen, and stored at −80°C until RNA extraction. RNA-seq was performed by Shanghai Majorbio Pharm Technology, Shanghai, China, on an Illumina HiSeq × 4000 instrument (2 × 150 bp read length). Data were analyzed on the Majorbio I-Sanger Cloud Platform (https://www.i-sanger.com). Gene transcription levels were quantified with RSEM, a software package for estimating gene and isoform expression levels from RNA-seq data, and normalized as transcript per million (TPM) values (Li et al., 2010; Li and Dewey, 2011). Analysis of differential gene expression was performed with the DESeq2 software package (Love et al., 2014). Multiple testing for false discovery rate (*p*-adjust) was calculated using the Benjamini–Hochberg method (Benjamini and Hochberg, 1995).

To corroborate RNA-seq data, the transcript abundance of four critical genes, namely, *lytD, lytE, cidA, cidB*, was quantified by qRT-PCR using *recA* as a housekeeping gene to normalize expression data. The primers used in these experiments are listed in Supplementary Table 1. Total RNA was extracted using the E.Z.N.A.TM Bacterial RNA Kit (Omega, USA). Purified RNA, after DNase I digestion, was quantified using a NanoDrop 2000c UV-vis spectrophotometer (Thermo Fisher Scientific, USA), and RNA integrity was assessed by agarose gel electrophoresis. Reverse transcription was performed using the Evo M-MLV RT Premix for qPCR (Accurate Biotechnology, China). Reactions were performed in triplicate on an ABI 7500 Real-Time PCR System (Applied Biosystems) using KAPA SYBR FAST Universal qPCR Kit (KAPA Biosystems). The comparative Ct (2^−ΔΔCt^) method was used to calculate the relative expression.

### Analytical methods

Bacterial growth was monitored by measuring OD_600_ on a spectrophotometer (model UV-7200, UNICO, Shanghai, China). HA concentrations were determined using 8-quinolinol according to a previously described protocol (Frear and Burrell, 1955).

## Results

### *Alcaligenes ammonioxydans* HO-1 antagonized other bacteria *via* producing extracellular HA

**Figure 1:**
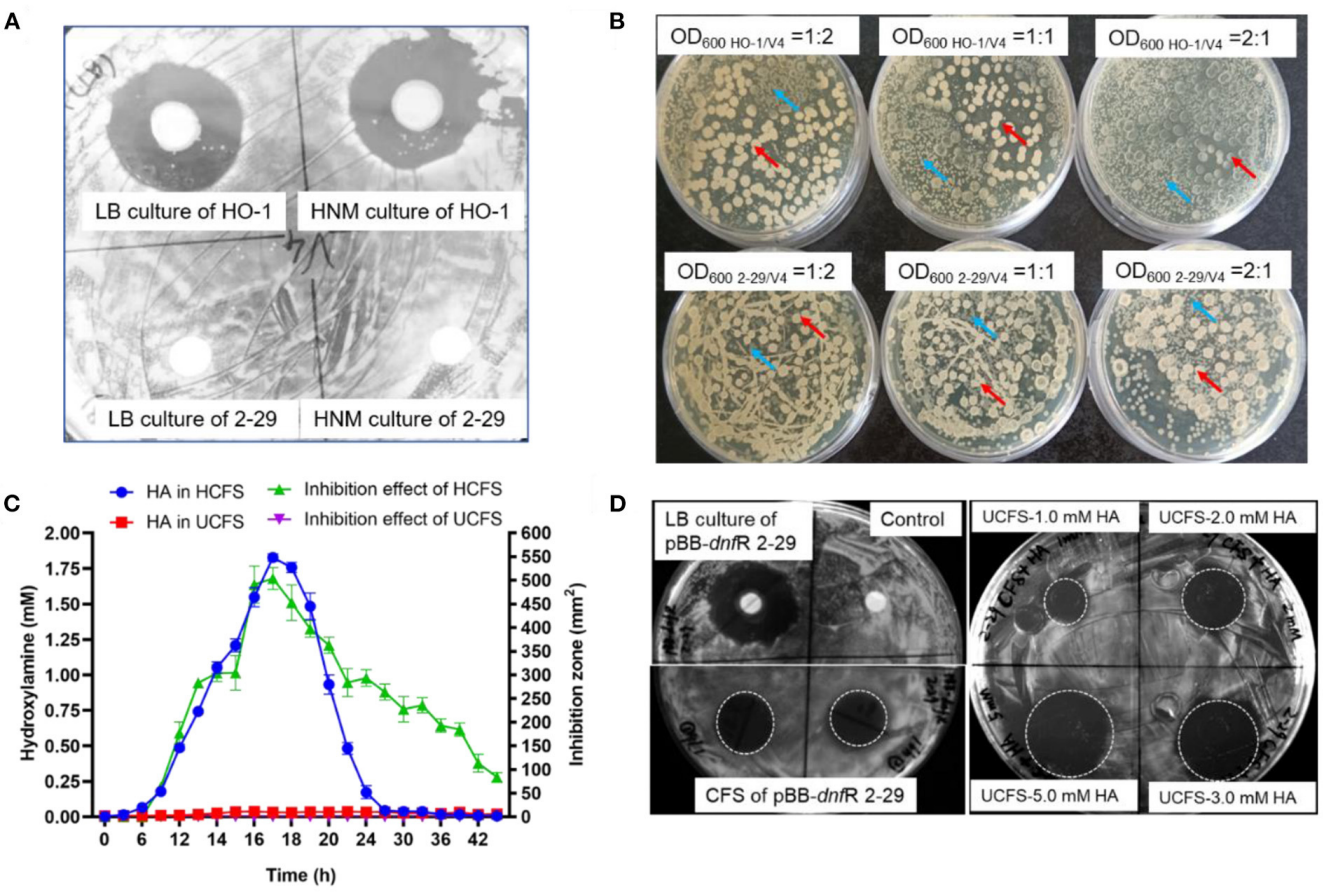
Contact-independent interbacterial antagonism of *A. ammonioxydans* HO-1 toward *B. velezensis* V4. **(A)** Comparison of antagonistic behavior between *A. ammonioxydans* HO-1 and 2-29, the latter failing to inhibit *B. velezensis* V4. **(B)** *A. ammonioxydans* HO-1 showed more competitive advantage when co-cultured with *B. velezensis* V4 with the initial OD_600_ ratios of 1:2, 1:1, and 2:1, respectively. Red arrow indicates *B. velezensis* V4 colonies, and blue arrow indicates *A. ammonioxydans* HO-1 or 2-29 colonies. **(C)** HA production and inhibition effect of HCFS/UCFS (CFS of *A. ammonioxydans* HO-1/2-29) at different time points in culture. Error bar represents standard deviations of three replicates. **(D)** The inhibitory capacity of the complementation strain of *A. ammonioxydans* 2-29 (pBB-*dnfR*) and UCFS containing specified HA concentrations show inhibition capacity toward *B. velezensis* V4.

We first investigated the inhibition of the growth of *B. velezensis* V4 by two different *Alcaligenes* strains. Preincubated V4 liquid cultures were spread on LB plates and HO-1 cultures pipetted onto a sterile disk in the middle of the dish. Under these conditions after 24 h of incubation, a large clear zone was formed around this disk, suggesting that HO-1 exhibited severe antagonism against V4 (Figure 1A). However, the presence of the HA-negative mutant *A. ammonioxydans* strain, 2-29, did not result in the formation of a clear zone, indicating that strain 2-29 could not antagonize V4 cell growth (Figure 1A).

Competition between the two *Alcaligenes* strains and *Bacillus* V4 was further investigated in co-culture experiments. Supplementary Figure 1 shows the growth curve of HO-1, 2-29, and V4 stains when cultured alone in liquid LB. In co-culture experiments, *Alcaligenes* HO-1 or 2-29 were plated together with V4 at initial OD_600_ ratios of 1:2, 1:1, and 2:1, respectively. The numbers of *B. velezensis* and *A. ammonioxydans* cells at the same OD_600_ differ due to their distinct cell size. The actual number of cells, as determined by colony-forming units, is ~2.5-fold for *A. ammonioxydans* than for *B. velezensis*. As shown in Figure 1B, HO-1 and 2-29 formed small colonies, while V4 formed big, white colonies on LB agar, making it easy to distinguish them during co-culture. At an initial OD_600_ ratio of 1:2, V4 cells established a broader spatial niche than those from HO-1. However, when the OD_600_ ratio increased to 1:1 and 2:1, HO-1 cells appeared to have a significant growth advantage. At 2:1 OD_600_ ratio, HO-1 colonies occupied almost the entire spatial niche, with only a few distorted *Bacillus* colonies surviving, with abnormal colony morphology. However, when HA-negative 2-29 cells were co-cultured with V4, the latter permanently established a broader spatial niche, irrespective of the initial OD_600_ ratio. These results indicated that, compared to the 2-29 mutant, HO-1 cells possess an interbacterial competitive advantage when coexisting with the otherwise robust *Bacillus* species.

Figure 1C illustrates the concentration of HA and the antagonistic growth-inhibiting effects of the HCFS and UCFS. In the culture media of HO-1 cells, HA concentration increased to almost ~2.0 mM between the 9th to 21st h, while 2-29 did not produce HA. The curve showing the inhibitory effect of HCFS exhibited an almost identical pattern. The peak in inhibitory capacity of HCFS at 21 h incubation time coincided with maximal concentration at this time point. Our previous study with HO-1 cells showed that the decrease in extracellular HA after the initial exponential growth phase was due to its conversion into nitrogen (Wu et al., 2021; Hou et al., 2022). HA was absent from UCFS, consistent with the lack of inhibitory action of 2-29 cells on V4 cell proliferation. The inhibitory effect of HCFS on V4 cells clearly indicated a contact-independent mechanism of antagonism. Given that the 2-29 strain, unable to produce HA due to a mutation, did not exhibit inhibitory capacity, it was reasonable to assume that HO-1 antagonized V4 primarily by producing HA. When the mutated 2-29 cells were transfected with a plasmid carrying the intact *dnfR* gene, the resulting pBB-*dnfR* 2-29 strain regained the ability to inhibit V4 cells, further supporting the role of HA in the contact-independent antagonism. Supplementing UCFS with HA at 2.0 mM concentration (UCFS-2.0 mM HA) also restored the inhibition (Figure 1D), and further increases in HA concentration resulted in additional gains in inhibitory effectiveness (UCFS-3.0 mM HA and UCFS-5.0 mM HA). Together, these results suggested an HA-mediated interspecies antagonism, a novel function of this compound that has not been observed previously.

It may be of note that the antagonism still existed after 24 h of culture period when the HA concentration in HCFS started to drop and was eventually reduced to zero. This observation suggests that alternative mechanism(s) of antagonism were likely to exist, particularly at later stages of the co-culture period. Therefore, an antagonistic effect of HO-1 caused by the existence of multiple metabolites cannot be excluded. However, during the first ~24 h of the antagonism, HA clearly acts as the key inhibitor in the interspecies competition.

To determine the antagonistic spectrum of HO-1 cells, 40 bacterial strains (Table 1) were tested by individually co-culturing them with HO-1. These experiments showed intense antagonistic effects against 33 laboratory-isolated and preserved strains, including *Bacillus* sp. (10 strains), *Pseudomonas* sp. (9 strains), *Vibrio* sp. (2 strains), *Comamonas* sp. (2 strains), *Rhodococcus* sp. (2 strains), *Escherichia coli, Enterobacter* sp., *Arthrobacter* sp., *Marinobacter* sp., *Exiguobacterium* sp., *Brevundimonas* sp., *Lysinibacillus fusiformis*, and *Castellaniella* sp. (Supplementary Figure 2). In contrast, no antagonism was demonstrable against seven closely related *Alcaligenes* strains (Supplementary Figure 2). Therefore, HO-1 exhibited a wide antagonistic spectrum, including Gram-positive and Gram-negative bacteria, while members of the *Alcaligenes* genus were resistant to inhibition.

### Characterization of the inhibitory effect of HA

**Figure 2:**
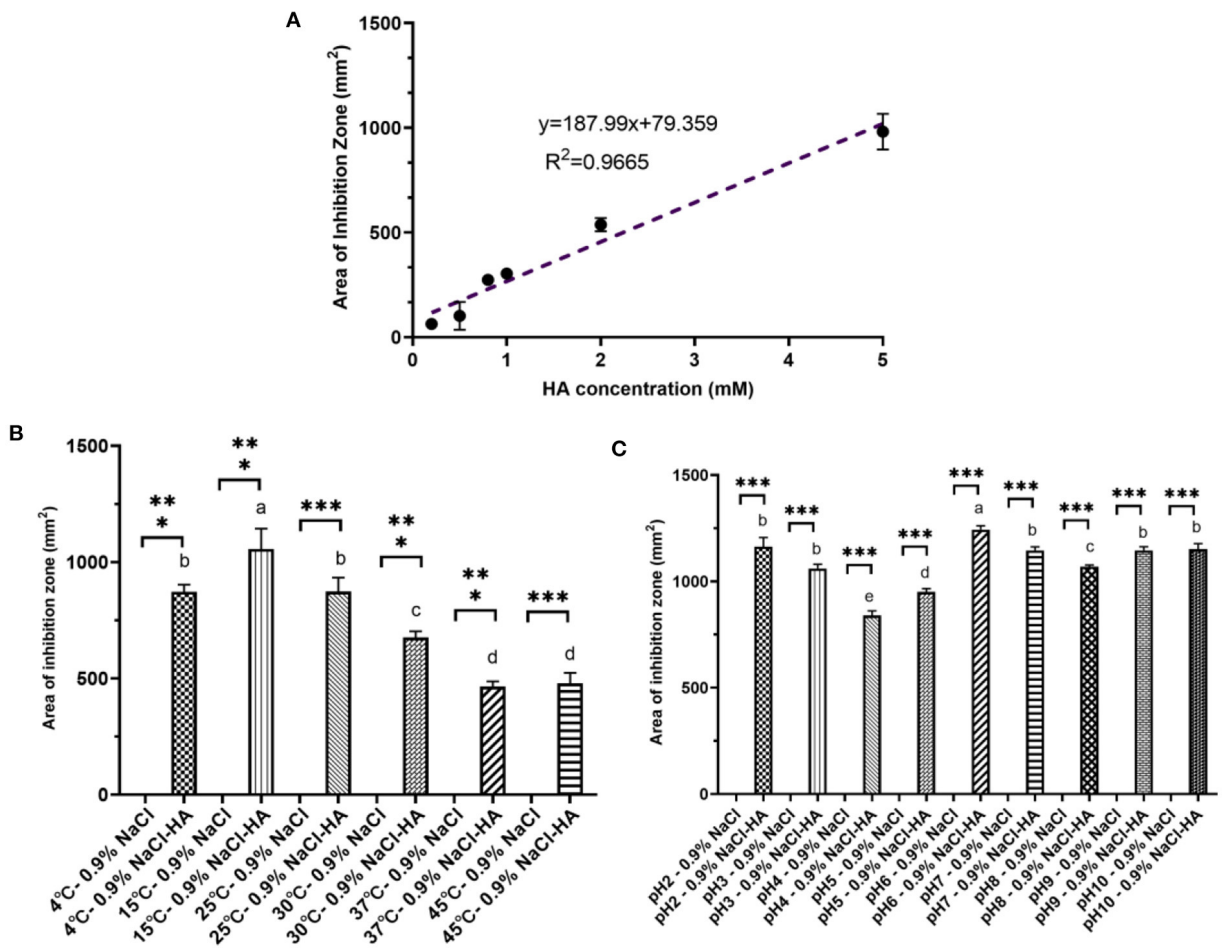
Characteristic of hydroxylamine inhibition toward *B. velezensis* V4. **(A)** Linear dose-dependency of HA-mediated inhibition. **(B)** The effect of temperature on HA-mediated inhibition. Pairwise comparisons between treatments are shown using a letter-based representation. Different letters indicate distinct significant differences. **(C)** The influence of pH on HA inhibition. Error bars represent standard deviation based on three replicates. Pairwise comparisons between treatments are shown using a letter-based representation. Different letters indicate distinct significant differences. *** means there is a significant difference between respective control and treatment.

To characterize the inhibitory effect of HA further, the influence of varying concentrations, temperatures, and pH was tested. In accordance with the observations shown in Figure 1C, the inhibitory capacity of HA was concentration-dependent. The inhibition zone forming around Oxford Cups gradually increased from 63.25 ± 0.58 mm^2^ at 0.2 mM to 537.79 ± 31.52 mm^2^ at 2.0 mM and to 981.25 ± 85.31 mm^2^ at 5.0 mM (Figure 2A). The effect of increasing HA concentrations at the beginning of inoculation affecting the lag phase of the growth curve of V4 grown in liquid LB was also tested. The lag phase of V4 gradually lengthened with increasing HA concentrations. The inoculated cells were completely inhibited and failed to grow within 30 h when the initial HA concentration exceeded 0.5 mM (Supplementary Figure 3).

When cultures were incubated at 4, 15, 25, 30, 37, and 45°C after the addition of 200 μl HA (2 mM), the area of inhibition zone produced was 872.40 ± 30.37, 1,056.61 ± 87.37, 872.92 ± 59.83, 675.62 ± 26.74, 464.98 ± 22.21, and 478.33 ± 45.32 mm^2^, respectively (Figure 2B). Thus, HA had the most substantial inhibitory effect at 15°C, and although its effectiveness decreased with rising temperatures, a robust inhibition could still be observed even at 45°C. This decline in inhibitory efficiency seen at higher temperatures may be due to the decomposition of HA (Lewis and Sax, 1996; Iwata et al., 2003).

Unlike temperature, pH did not significantly affect the area of inhibition zone produced by 200 μl HA (2 mM). The zone was 1,193.99 ± 42.74 mm^2^ at pH 2.0, 1,074.67 ± 20.41 at pH 3.0, 854.87 ± 21.79 at pH 4.0, 961.63 ± 15.46 at pH 5.0, 1,256.00 ± 17.68 at pH 6.0, 1,133.54 ± 16.96 at pH 7.0, 1,074.67 ± 8.20 at pH 8.0, 1,133.54 ± 17.35 at pH 9.0, and 1,133.54 ± 25.92 mm^2^ at pH 10.0 (Figure 2C).

### HA induced the autolysis and aggregation of exponentially growing *B. velezensis* V4 cells

**Figure 3:**
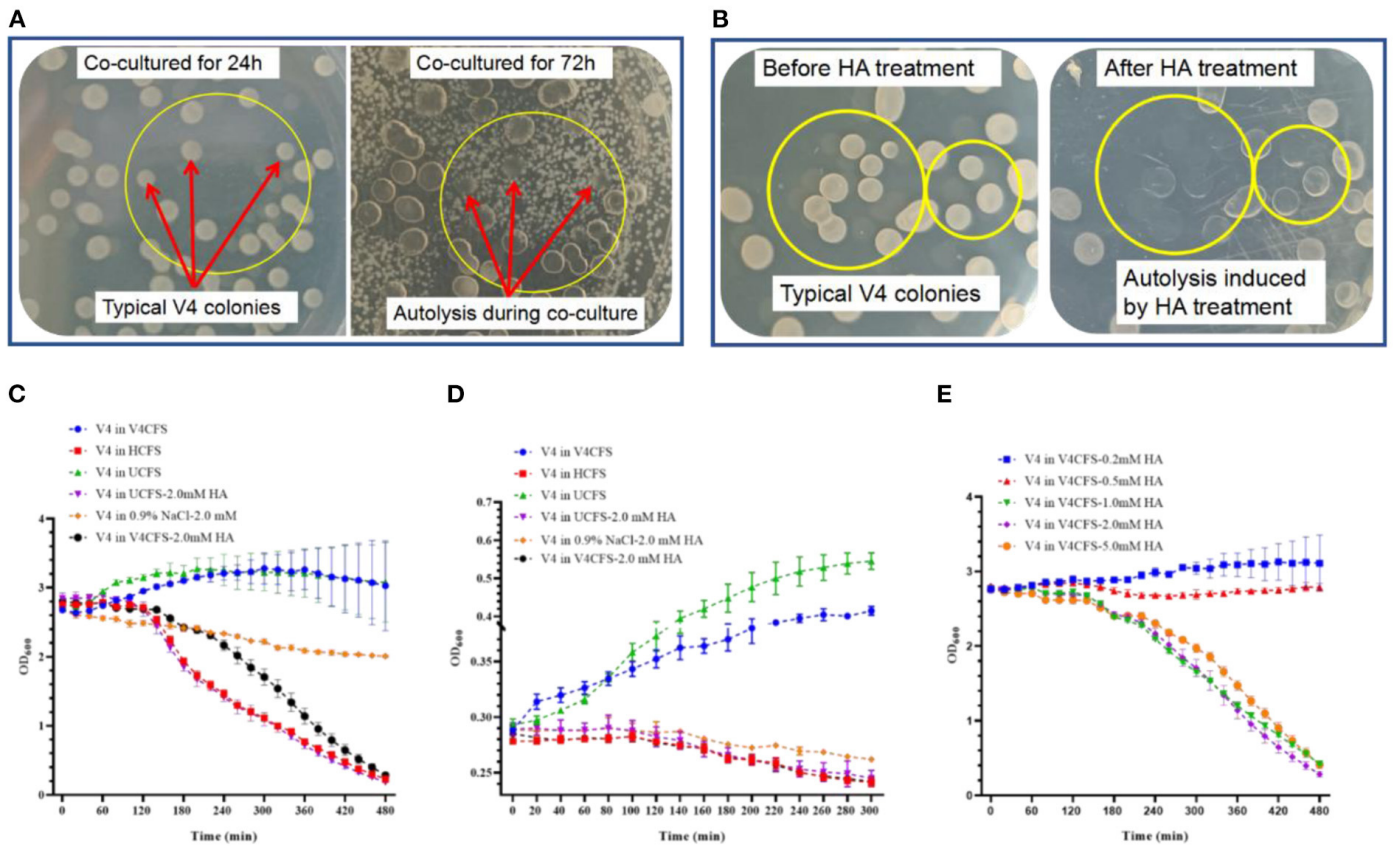
The effect of HO-1 co-culture or treatment with HA on the colony morphology and survival kinetics of *B. velezensis* V4. **(A)** Changing appearance of *B. velezensis* V4 colonies during co-culture with *A. ammonioxydans* HO-1. Red arrows inside yellow circles indicate typical *B. velezensis* V4 colonies before significant *A. ammonioxydans* HO-1 growth at 24 h (on the left) and to the same colonies after the growth of *A. ammonioxydans* HO-1 caused autolysis at 72 h (right). **(B)** Typical *B. velezensis* V4 colonies before the treatment with HA (left) and the same plaques following autolysis after HA treatment (right). **(C)** Survival kinetics of *B. velezensis* V4 (high initial population density, OD_600_ = 3.0) in V4CFS (as control), HCFS, UCFS, UCFS-2.0 mM HA, 0.9% NaCl-2.0 mM HA, V4CFS-2.0 mM HA. **(D)** Survival kinetics of *B. velezensis* V4 (low initial population density, OD_600_ = 0.3) in V4CFS (as control), HCFS, UCFS, UCFS-2.0 mM HA, 0.9% NaCl-2.0 mM HA, V4CFS-2.0 mM HA. **(E)** Survival kinetics of *B. velezensis* V4 in V4CFS containing 0.2, 0.5, 1.0, 2.0, and 5.0 mM HA. Error bars represent standard deviations based on three replicates.

The inhibitory zone seen in Figure 1A and the prolongation of lag phase shown in Supplementary Figure 3 indicated direct toxicity or inhibition caused by HA. Moreover, HA appeared to have different effects on rapidly growing and already established colonies. The abnormal morphology of V4 colonies during co-culture with HO-1 is clearly shown in Figure 1B. With the extension of co-cultivation time, existing normal colonies of V4 disappeared gradually, leading to the eventual appearance of lysed plaques. As shown in Figure 3A, during co-cultures on LB plate V4 cells initially formed complete and normal colonies by 24 h. However, these underwent lysis as HO-1 colonies grew, with clear lytic plaques forming by 72 h. These observations implied that the presence of HO-1 cells induced the lysis of both proliferating and preexisting bacteria. To confirm that this phenomenon was due to the presence of HA produced by HO-1, we treated existing V4 colonies with 2.0 mM HA. As shown in Figure 3B, this resulted in the formation of lytic plaques of colonies seen at the late stages of co-culture experiments (Figure 3B). Therefore, lysis of both actively proliferating and established V4 colonies could be induced by co-culturing them with HO-1 or exposing them to an equivalent amount of HA.

To establish some of the mechanisms leading to the lysis of V4 cells, the survival kinetics of exponentially growing V4 cultures were studied under different culture conditions.

Figures 3C,D show the survival kinetics of V4 cells grown in V4CFS (as control), HCFS, UCFS, and 2.0 mM HA, starting at a high and low initial population density (OD_600initial_ = 3.0 and 0.3). As expected, V4 grew normally in V4CFS and UCFS with OD_600_ values steadily increasing. However, at both high and low initial densities, the biomass decreased sharply after being exposed to HCFS, UCFS-2.0 mM HA, or V4CFS-2.0 mM HA. Interestingly, the biomass only showed a relatively modest decline when the cells were kept in 0.9% NaCl-2.0 mM HA. This rapid collapse of the V4 cell population in response to HA in the presence of nutrient-rich media suggested an energy-dependent autolysis of cells, while the slow decline in HA in NaCl was compatible with direct toxicity. To explore the role of nutritional factors further, we exposed V4 cells to HA in LB and MM medium and 0.9% saline. Survival kinetics in LB with and without HA were almost identical to those seen in V4CFS. In MM, the biomass of V4 increased slightly. This may be explained by a lag phase resulting from changes in the medium. HA in MM resulted in a continuous decline in OD_600_, but this happened at a slower rate compared to the decline seen in LB containing HA (Supplementary Figure 4).

The dose dependence of the HA-mediated cell number decline is shown in Figure 3E. Survival kinetics of V4 in V4CFS showed a small dose-dependent decline at 0.2 and 0.5 mM HA concentration. At 1.0 mM, the biomass dropped drastically, with no further significant effect seen at 2.0 and 5.0 mM HA concentrations.

**Figure 4:**
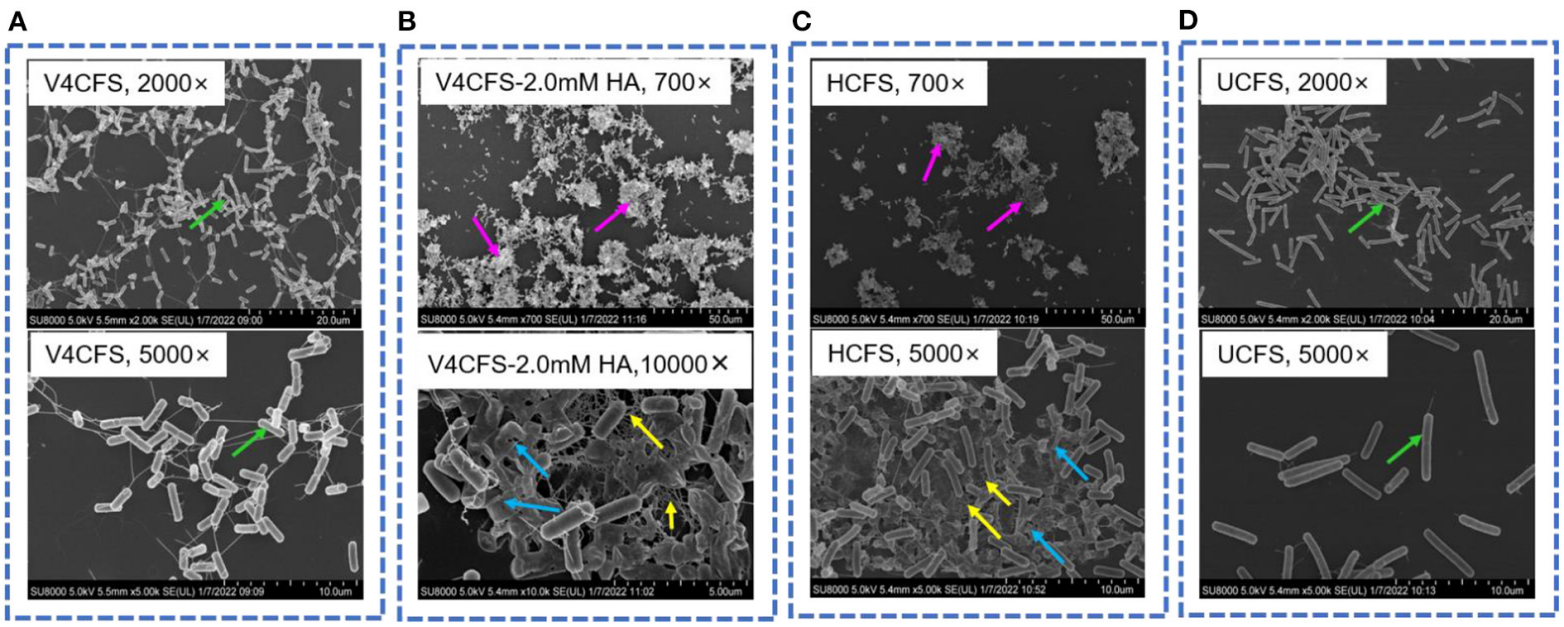
Morphology of *B. velezensis* V4 under different culture conditions. Cell morphology of *B. velezensis* V4 in V4CFS (**A**, 2,000× and 5,000×), V4CFS-2.0 mM HA (**B**, 700× and 10,000×), HCFS (**C**, 700× and 5,000×), UCFS (**D**, 2,000× and 5,000×) at 180 min. Green arrows indicate normal morphology of cells with flagellum, purple arrows indicate cell aggregation, blue arrows indicate cell lysis and cellular debris, and yellow arrows indicate biofilm analogs.

Next, we assessed the impact of HA exposure on cell morphology. V4 cells growing in V4CFS appeared normal with flagellum and showed an orderly distribution (Figure 4A). Exposure to HA for 3 h caused pronounced cell aggregation, the loss of motility-related features, and cell wall lysis (Figure 4B). HA appeared to promote biofilm formation by *B. velezensis*. Cell lysis, the release of intracellular content, and cellular debris facilitate cell aggregation, leading to the formation of biofilm analogs. Cells grown in HCFS showed the same morphology observed as in V4CFS-2.0 mM HA, with aggregation and cell lysis (shown in Figure 4C), while in UCFS cells remained ordered without aggregation or lysis (Figure 4D).

### Changes in the expression of genes modulating multicellular behavior in *B. velezensis* V4 in response to HA

**Figure 5:**
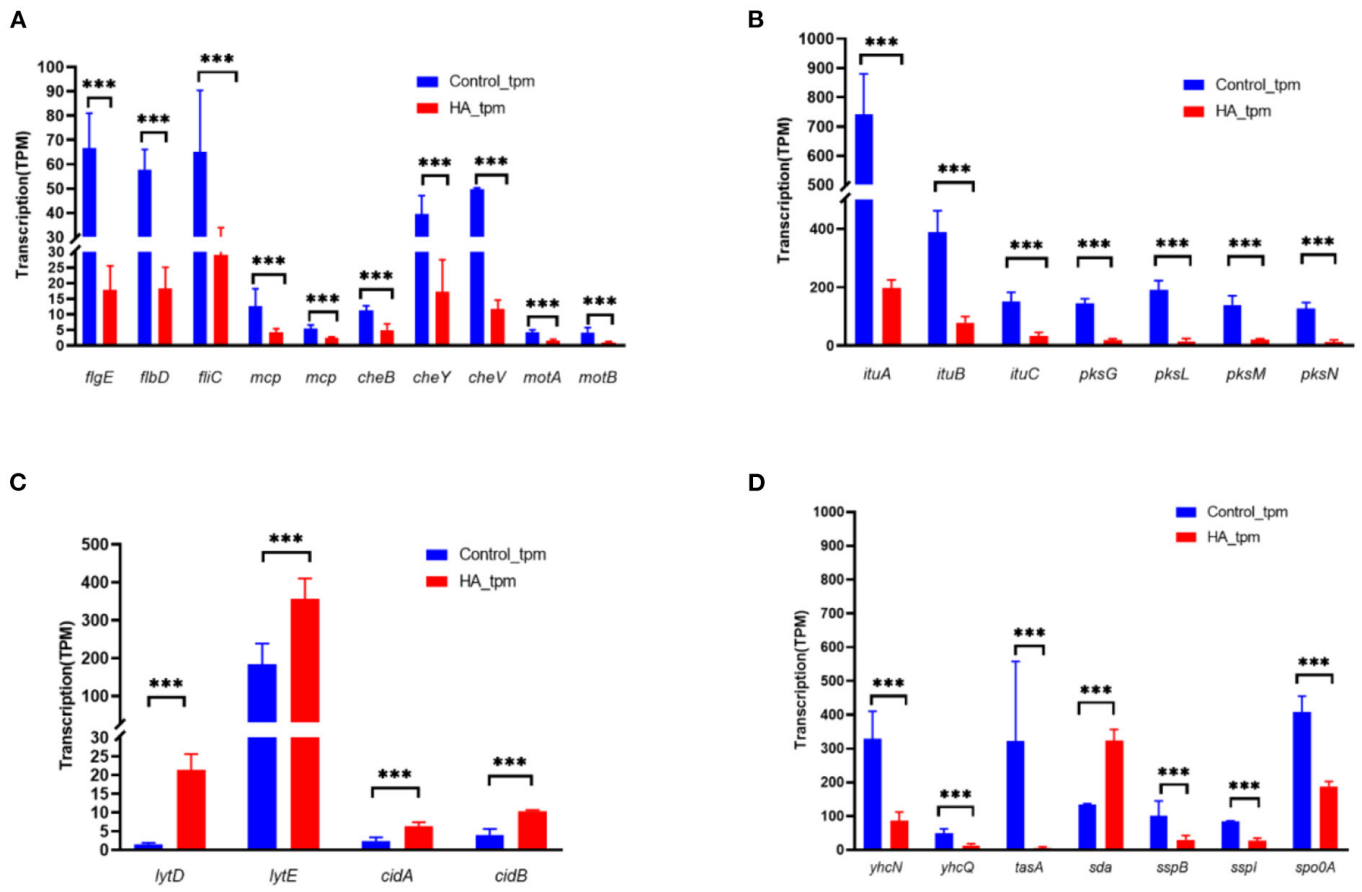
Changes in the abundance of genes modulating multicellular behavior in *B. velezensis* V4 upon exposure to HA. **(A)** Expression of motility and chemotaxis-related genes in *B. velezensis* V4 upon exposure to hydroxylamine. **(B)** Expression of polyketide synthetase (PKS) and non-ribosomal peptide synthetase (NRPS) genes in *B. velezensis* V4 upon exposure to hydroxylamine. **(C)** Expression of autolysis-related genes in *B. velezensis* V4 upon exposure to hydroxylamine. **(D)** Expression of sporulation-related genes in *B. velezensis* V4 upon exposure to hydroxylamine. RNA-seq data were normalized to TPM. Error bars indicate standard error based on three replicates. *p*-values were calculated using a negative binomial distribution-based test, and false discovery rate (*p*-adjust) was calculated using the Benjamini/Hochberg method. At each time point, ***represents a significant change in transcript abundance (FDR < 0.05).

Gene expression profiling using RNA-seq was performed to identify changes associated with exposing V4 cells to HA. As shown in Figure 5A, genes involved in motility and chemotaxis showed significantly reduced transcription as a result of HA treatment. Genes necessary for the formation assembly of the flagellar body, such as *flgE* (flagellar basal body rod protein FlgG), *flbD* (flagellar protein FlbD), and *fliC* (flagellin), were all downregulated, with log_2_fc values of −1.79, −1.49, and −1.04 compared to the control sample. A similar reduction was seen in the abundance of genes involved in chemotaxis, including *mcp* (methyl-accepting chemotaxis protein), *cheB* (chemotaxis response regulator protein), *cheY* (chemotaxis protein CheY), and *cheV* (chemotaxis protein CheV).

HA also influenced the expression of genes involved in secondary metabolism. Both *ituA-C* (iturin family lipopeptide synthetase A, B, and C) and *pksG, L-N* (polyketide synthase PksG, PksL, PksM, and PksN) showed significantly reduced transcription in the presence of HA (Figure 5B).

The genome of V4 encodes two families of cell wall-lytic enzymes. The peptidoglycan DL-endopeptidase family comprises four primary cell wall-lytic autolysins (LytC, LytD, LytE, and LytF). The antiholin-like murein hydrolase family comprises CidA and CidB. The abundance of *lytE* transcripts was significantly increased (>12-fold) in the presence of HA (Figure 5C), and *lytD*, c*idA*, and *cidB* were also significantly upregulated (>2-fold). These gene expression changes are compatible with the morphological alterations described in the “HA induced the autolysis and aggregation of exponentially growing *B. velezensis* V4 cells” section, supporting the observations that exposure to HA or the co-culture of V4 cells with HO-1 led to the degradation of motility-related structures while promoting autolysis.

Sporulation-related genes, including *yhcN* (sporulation lipoprotein), *yhcQ* (spore coat protein), *tasA* (spore coat-associated protein N), *sspB* and *sspI* (small acid-soluble spore protein B and I), *spo0A* (stage 0 sporulation protein A, a response regulator involved in a two-component system), all showed significantly reduced transcription in the presence of HA (Figure 5D). In contrast, the inhibitory *sda* gene, encoding the sporulation histidine kinase inhibitor A, was significantly upregulated. These differences in gene expression indicated that the process of sporulation was significantly inhibited in the presence of HA.

To validate the above gene expression data, qRT-PCR was carried out to evaluate the abundance of *lytD, lytE, cidA*, and *cidB* transcripts in HA-treated and control cells. The results of qRT-PCR (see Supplementary Figure 5) strongly supported the changes detected by RNA-seq. mRNAs encoding *lytD, lytE, cidA*, and *cidB* were upregulated by 22.95-, 4.39-, 2.13-, and 2.8-fold, according to amplification results, whereas during RNA-seq the same genes were detected as upregulated 13.92-, 2.06-, 2.64-, and 2.63-fold.

## Discussion

During attempts to establish a potent bacterial consortium for the control of other infections, a contact-independent interbacterial antagonism was detected between *A. ammonioxydans* HO-1 and *B. velezensis* V4. We found ample evidence that the production of HA was responsible for this antagonism. The HA-negative mutant strain, *A. ammonioxydans* 2-29, showed no antagonism and reconstituting this mutant with the intact *dnfR* gene restored the ability to inhibit the growth of V4 cells, indicating that HA was the key molecule in this process. Since other *Alcaligenes* species are potential candidates in the production of antibacterial substances (Felestrino et al., 2020; Fernandes et al., 2021), it is tempting to speculate that other antagonistic secondary metabolites existed. The degradation of HA beyond ~24 h of culture, while some antagonism could still be observed beyond this time point suggested that this may be the case especially during the late growth stage of HO-1. Nonetheless, HA production was clearly the primary cause of antagonism described in this article. The inhibitory effect of HA on *Bacillus* cells under various conditions appeared relatively stable under a range of temperatures and pH.

*Alcaligenes ammonioxydans* HO-1 showed antagonistic behavior against various microorganisms and had obvious interspecies competition advantages when co-cultured with the majority of the 40 strains tested. It was previously reported that *A. faecalis* and *Alcaligenes viscolactis* can target Gram-positive bacteria outside the *Staphylococcus* genus, as well as certain Gram-negative species and *Candida glabrata* (Fuqua, 2020). However, the broad-spectrum antagonism observed in those experiments was contact-dependent. It is of note that HO-1 did not have an antagonistic effect on closely related *Alcaligenes* species. *Alcaligenes* strains typically produce HA during nitrogen metabolism; consequently, they may have developed strategies for converting HA to other non-toxic substances (Tsujino et al., 2021; Wu et al., 2021).

Typically established colonies of V4 showed a trend to disappear gradually, eventually leading to the appearance of lysed plaques when co-cultured with HO-1 or exposed to HA. We observed that the lysis of V4 cells following HA exposure only occurred in the presence of nutrients. Exposure to HA in the absence of nutrients, in 0.9% NaCl, did not cause the same cell lysis, indicating an energy-dependent active population response. The upregulated expression of autolysis-related genes shortly after HA treatment confirmed this assumption. Furthermore, the effect of HA was only dose-dependent in the 0.2–2.0 mM concentration range, with the most drastic change occurring between 05 and 1.0 mM. Treatment with higher concentrations of HA did not cause a more rapid or profound population decline. This also prompted us to distinguish between an inhibition, a toxic effect, and a trigger effect when HA was acting as a possible mediator of antagonism.

Exposure to HA induced autolysis and aggregation in *Bacillus* cells. Autolysis is a form of population-level behavior characterized by self-digestion of the cell wall, resulting in complete cellular disintegration. This benefits the surviving cell population by the provision of nutrients and the transfer of genetic material. It represents a cooperative behavior promoting biofilm development (Diggle et al., 2007). Autolysis has been linked directly to quorum sensing in some species (McGivney, 2017). In addition, autolysis at low population density (OD_600__initial_ = 0.3) was less pronounced compared to that seen at a high population density (OD600 initial = 3.0). This led us to speculate that the *Bacillus* cells may respond to HA in a quorum-sensing manner. As both *Bacillus* and *Alcaligenes* strains live in the soil, it is plausible that *B. velezensis* have developed a “tune sensing” mechanism to recognize *Alcaligenes* by detecting its HA production. This hypothesis is supported by the range of genes showing differential expression as HA was modulating multicellular behavior.

On the basis of these results, a number of specific mechanisms may explain the effects of HA when antagonizing *B. velezensis* V4. (1) The gradual lengthening of the lag phase with increasing HA concentrations indicates an inhibitory action. (2) The relatively small population decline when the V4 cells were exposed to HA in 0.9% NaCl-2.0 mM HA, with only a 17.8% reduction in cell numbers, is consistent with direct toxicity and potential mutations. Mutations could have interesting consequences between the two coexisting bacteria. Since *Alcaligenes* produce HA during nitrogen metabolism, they must have strategies for converting HA to non-toxic substances to detoxify it (Tsujino et al., 2021; Wu et al., 2021). Could HA-induced mutations render V4 more resistant to this compound after a period of time in combined colonies? (3) A signal molecule for antagonism. As observed in Figure 3C, HA functions as a signaling molecule for exponentially growing *B. velezensis* cells in bulk (>0.5 mM). In this capacity, HA triggers a population-level response, causing autolysis. The observed overall antagonism of HO-1 cells against V4 is likely to represent the combined effect of these three specific mechanisms.

Transcriptome profiling and real-time quantitative PCR analysis provided further data supporting a population response of exponentially growing *B. velezensis* cells. Genes involved in motility and chemotaxis showed significantly reduced transcription in the presence of HA. These transcription-level observations were consistent with the cell aggregation and morphological changes affecting motility observed on SEM imaging.

Genes encoding polyketide synthetase and a non-ribosomal peptide synthetase showed significantly reduced transcription. These results explained why the robust V4 cells, normally exhibiting powerful protective mechanisms, could be inhibited by *Alcaligenes* strains or HA. In addition, genes involved in the sporulation process appeared to be significantly inhibited in the presence of HA. Sporulation plays a key role in cell fate decision in the life cycle of *Bacillus* (Hoch, 1993), and the inhibition of this process reduces the chances of survival through spore dormancy, giving *Alcaligenes* strains a survival advantage.

Chemotactic proteins belong to the two-component system (Garrity and Ordal, 1995). The process of sporulation is regulated by transcriptional control of its constituent proteins and by the regulation of phosphate flux (Hoch, 1993). Similarly, the process of autolysis is also controlled by cell signaling events (McGivney, 2017). It is interesting that the accumulation of HA caused by the presence HO-1 bacteria triggered auto-aggregation, autolysis, and biofilm formation by the V4 strain. Several recent studies suggested the importance of HA as a critical nitrogen metabolite in microbial interactions within microbial communities and engineered systems (Feng et al., 2021; Liu et al., 2021; Soler-Jofra et al., 2021; Zhao et al., 2021). However, the precise role of HA was poorly defined in these studies. The influence of HA on motility, chemotaxis, cell signaling, and even the two-component system indicates that it may function as a signaling molecule. Indole is a molecule with potentially comparable actions, receiving attention due to its diverse biological role in affecting a variety of bacterial strains (Lee and Lee, 2010). A high concentration of indole (~2 mM) was shown to decrease cell growth in *E. coli* and disrupt the bacterial envelope. Similar to indole, HA might function as an interspecies signaling molecule (Kumar and Sperandio, 2019; Zarkan et al., 2020). This hypothesis, while appealing, needs to be further investigated in other bacterial species and complex systems, including multispecies bacterial consortia.

Interbacterial antagonism can impact microbiome assembly and stability. It can be potentially exploited to modulate microbial communities in diverse environments, from natural habitats to industrial bioreactors (Yim and Wang, 2021). *B. velezensis* strains show remarkable efficacy in controlling various plant diseases, primarily due to their outstanding antimicrobial activity and ability to colonize plant surfaces (Luo et al., 2021). *Alcaligenes* species are another group of PGPR with proven beneficial activities. Thus, combining them in a beneficial consortium is an appealing theoretical possibility. However, interbacterial antagonism should be considered when assembling a consortium used for plant growth promotion. The presented findings remind us that the inclusion of *Bacillus* and *Alcaligenes* species should be approached with caution when attempting to assemble a beneficial bacterial consortium.

## Conclusion

This study demonstrated pronounced antagonism of *A. ammonioxydans* HO-1 exhibited against a wide range of bacterial genera, including *B. velezensis* V4. This broad-spectrum inhibitory capacity of HO-1 was due to the production of extracellular HA that induced autolysis and aggregation in *B. velezensis* and regulated multicellular behaviors. The influence of HA on motility and chemotaxis, cell signaling, and even the two-component system indicates that HA may function as a signaling molecule. This study is the first description of a novel inhibitory mechanism of *Alcaligenes* against *B. velezensis* and provides valuable references for optimizing the assembly of a biocontrol consortia for practical applications.

## Data availability statement

The datasets presented in this study can be found in online repositories. The names of the repository/repositories and accession number(s) can be found at: NCBI SRA - SRR18651101, SRR18651102, SRR18651103, SRR18651098, SRR18651099, and SRR18651100.

## Author contributions

X-YG developed the overall conceptual framework and wrote the initial draft. YL isolated the strain *A. ammonioxydans* HO-1 used in this work. WX and LM helped in screening the mutant strain *A. ammonioxydans* 2-29. Z-PL supervised all the experiments and revised the manuscript. All authors contributed to manuscript revision, read, and approved the submitted version.

## Funding

This study was supported by the China National Key R&D Program (2019YFA0905504), the Biological Resources Program, Chinese Academy of Sciences (KFJ-BRP-009-004), and the National Natural Science Foundation of China (Grant No. 91951101).

## Conflicts of interest

The authors declare that the research was conducted in the absence of any commercial or financial relationships that could be construed as a potential conflict of interest.

## Publisher's note

All claims expressed in this article are solely those of the authors and do not necessarily represent those of their affiliated organizations, or those of the publisher, the editors and the reviewers. Any product that may be evaluated in this article, or claim that may be made by its manufacturer, is not guaranteed or endorsed by the publisher.
