# Supplementary material for: Alcaligenes ammonioxydans HO-1 antagonizes Bacillus velezensis via hydroxylamine-triggered population response
Source: Front Microbiol. 2022 Jul 22;13:920052. doi: 10.3389/fmicb.2022.920052 (PMC9355588; doi:10.3389/fmicb.2022.920052)
Supplement: Supplementary file 1 [file Data_Sheet_1.PDF]

Frontiers in Microbiology-Microbial Symbioses

Supplementary materials for

Alcaligenes ammonioxydans HO-1 antagonizes other bacteria via extracellular hydroxylamine production triggering a population response in *Bacillus velezensis* V4

Xi-Yan Gao[1], Wei Xie[1,2], Ying Liu[1], Lan Ma[1,2], Zhi-Pei Liu[1*]

[1]State Key Laboratory of Microbial Resources, Institute of Microbiology, Chinese Academy of Sciences, Beijing 100101, China.

[2] University of Chinese Academy of Sciences, Beijing, China

* Correspondence: Institute of Microbiology

Chinese Academy of Sciences

No. 1 West Beichen Road

Chaoyang District, Beijing 100101

P. R. China

Tel: +10-64806081

Email: liuzhp@im.ac.cn

Supplementary Table S1. Genes and primers used in qRT-PCR

Genes	Forward primer	Reverse primer
lytD	5’-CCGCTCACTTATCAAATCTCAG-3’	5’-GTCAGTTCGGCAAGAAGATT-3’
lytE	5’- TCAAAGAAGCCTTCATCCAGC-3’	5’- AAGAAGACGAAGTCTCCTCTTGA-3’
cidA	5’- AGTCATACGCATCATCTTGC-3’	5’- TAATTGATGACGCCCGTCATG-3’
cidB	5’- ATGATCGGCGGACAATGGA-3’	5’- ACCATTCCGGAGAATAAACCGG-3’
recA	5’- TCGTCAGGCAGCCTTAGATAT-3’	5’- CGGTATCAAGTGCAAGGGAA-3’

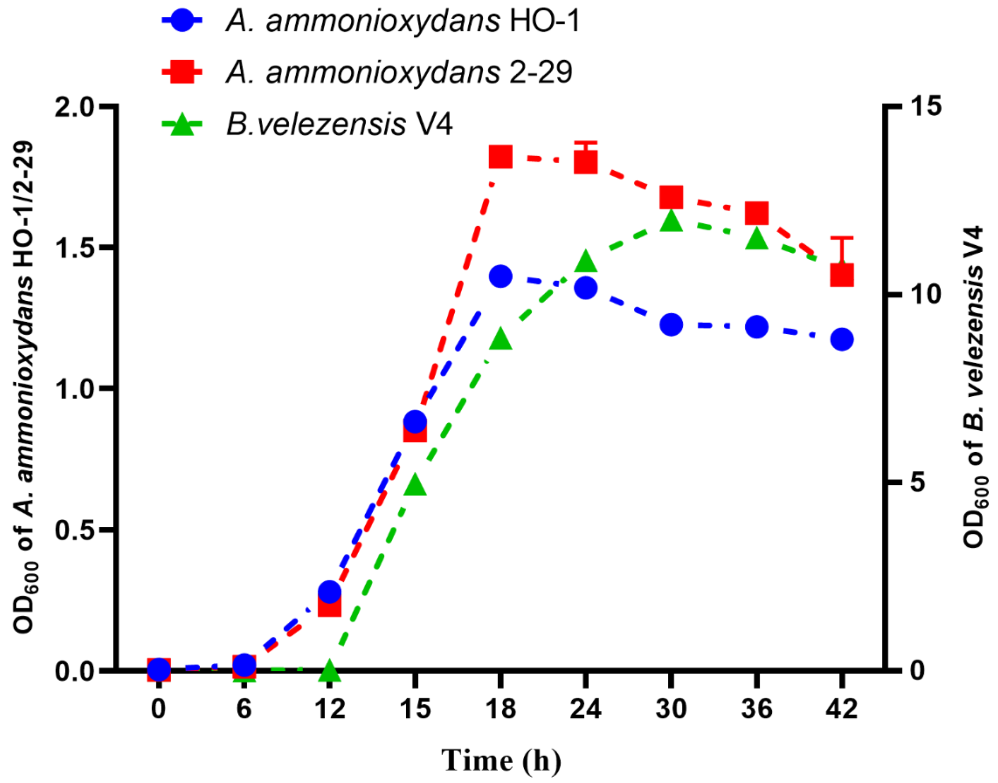

Supplementary Figure S1. Growth curve of *Alcaligenes ammonioxydans* HO-1/2-29 and *Bacillus velezensis* V4. Error bar represents standard deviations of three replicates.

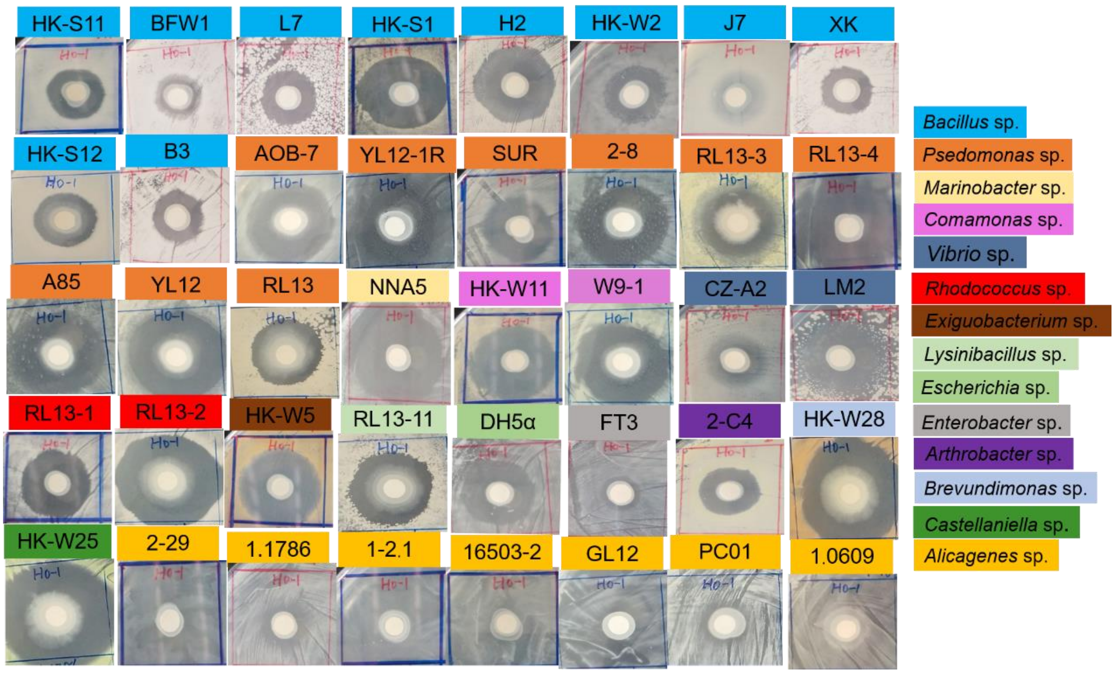

Supplementary Figure S2. *Alcaligenes ammonioxydans* HO-1 antagonizes broad-spectrum bacteria

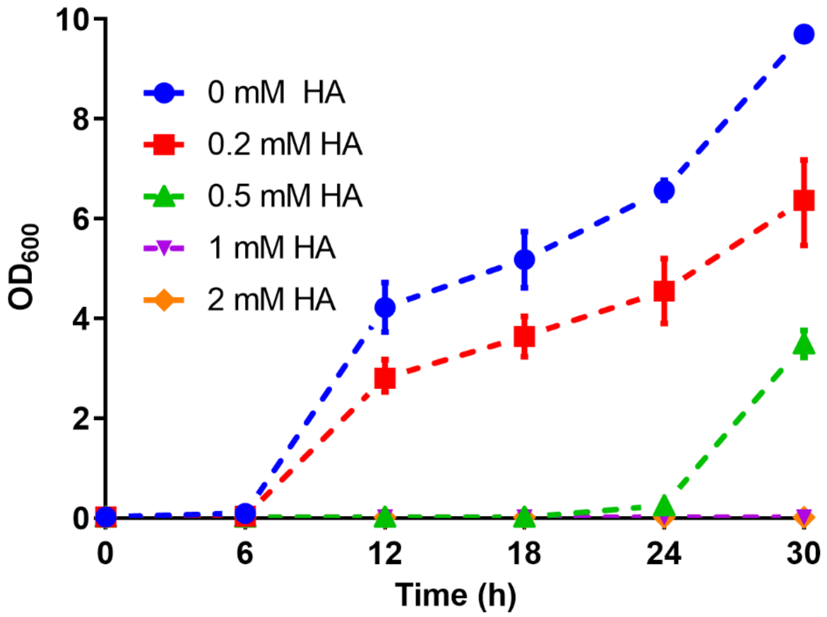

Supplementary Figure S3. The effect of HA on the growth curve of *B. velezensis* V4 in liquid LB. Error bar represents standard deviations of three replicates.

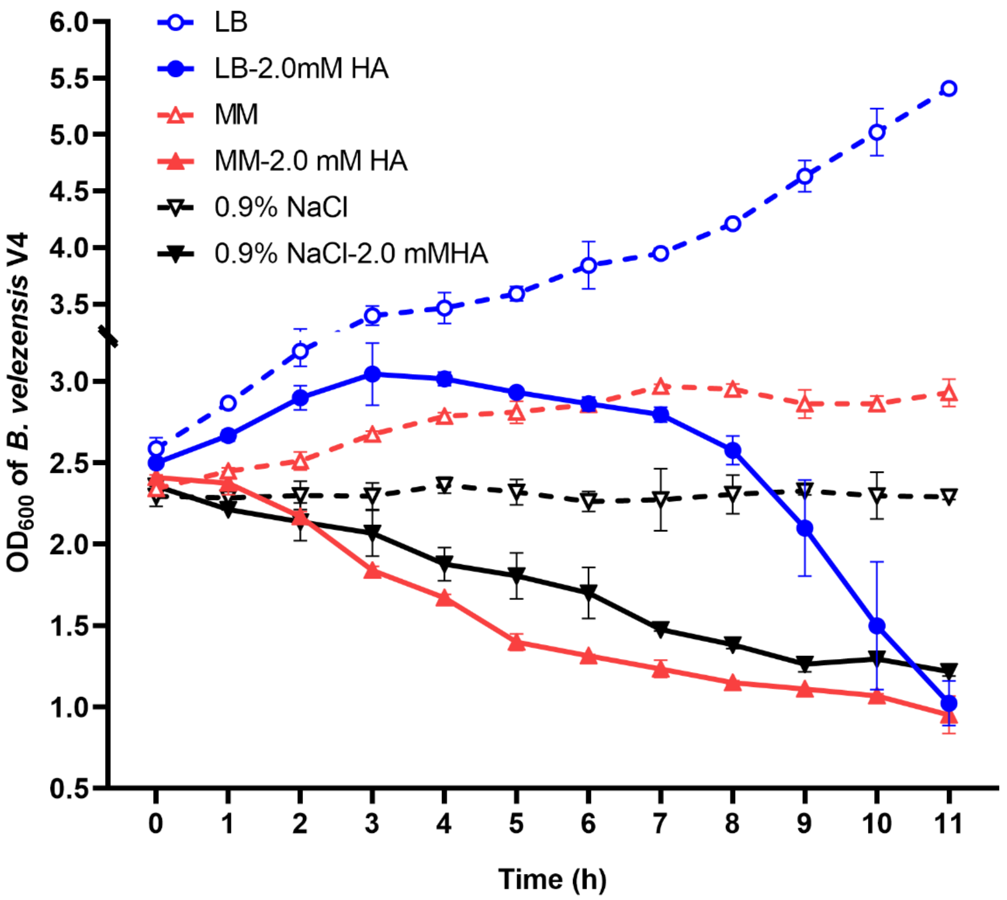

Supplementary Figure S4. Survival kinetics of *B. velezensis* V4 grown in LB/Mimimum medium/0.9% NaCl with and without HA. Error bars represent standard deviations based on three replicates.

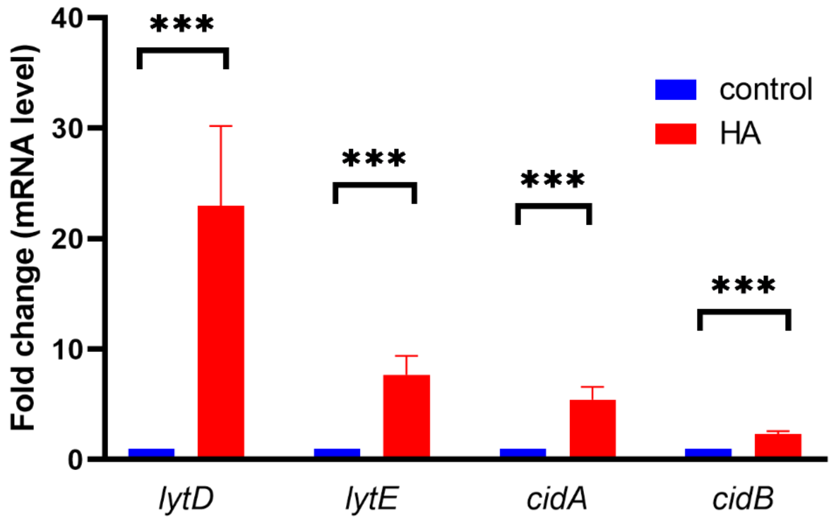

Supplementary Figure S5. Quantitative RT-PCR results to verify gene expression for *lytD*, *lytE*, *cidA*, and *cidB*. Error bars represent standard deviations based on three replicates.
